# Biopreservation and Reversal of Oxidative Injury During Blood Storage by a Novel Curcumin-based Gel Formulation

**DOI:** 10.21203/rs.3.rs-4277591/v1

**Published:** 2024-05-06

**Authors:** Wayne Hicks, Sirsendu Jana, Tigist Kassa, Richard Prince, Pedro Cabrales, Joel Friedman, Abdu I. Alayash

**Affiliations:** United States Food and Drug Administration; United States Food and Drug Administration; United States Food and Drug Administration; Vascarta Inc; University of California; Vascarta Inc; United States Food and Drug Administration

**Keywords:** Curcumin, VAS-101, Red blood cells, Fibrinogen

## Abstract

Blood storage lesion induces cytosolic and membrane changes driven in part by hemoglobin (Hb) oxidation reactions within red blood cells (RBCs). A novel gel formulation containing the antioxidant curcuminoids in a biocompatible solvent system was used to deliver curcumin into RBCs. Incubation of peroxide treated RBCs stored in PBS with curcumin gel led to a reduction in prooxidant ferrylHb and recovery in ATP. Curcumin treatment prevented band 3 tyrosine (Y359 and Y21) phosphorylation. RBCs stored in AS-3 solutions for 28, 35, 42 and 49 days, following a single-dose of 100μM curcuminoids at each time points, caused reduction in protein carbonylation and considerable recovery in ATP levels. Proteomic analysis revealed minimal changes in the proteomic landscape in 35 days. However, a downregulation in fibrinogen was observed in the treated samples which may reduce RBC aggregation. Additionally, we used a guinea pig model where the circulation of infused aged RBCs can be extended (approximately 10%) when treated with curcumin gel at the start of storage. Our data therefore provide mechanistic insights and supportive animal data into benefits of treating stored RBCs with a novel curcuminoid formulation based on the biopreservation of RBC membrane integrity, redox balance, and increased longevity in circulation.

## Introduction

During storage, RBCs undergo metabolic, oxidative, and physiological changes collectively described as the “storage lesion(s)” which can impact both therapeutic efficacy and circulation lifetime of transfused RBCs^1^. RBCs are typically stored in a variety of preservation solutions at 4–6 °C to minimize the extent of changes during storage. Oxidative as well as metabolic changes in Hb and other RBC proteins were reported to play a key role in overall physiological and clinical consequences of the storage lesion^2^. The progression of the storage lesion was shown recently to be driven in part by internal redox oxidative reactions, mainly Hb’s oxidative side reactions, hence the process is also termed “oxidative lesion”^3^.

Current blood banking practices in the United States involve the refrigerated storage of blood for up to 42 days. However, RBCs begin to undergo a progressive reduction in flexibility and oxidative stability which may alter blood flow and result in a decrease in tissue oxygenation. A decrease in RBCs deformability and stability results in an enhanced propensity for hemolysis and for production of pro-inflammatory microparticles(MPs)^4^. Hemolysis, in turn, releases Hb which in turn scavenges nitric oxide (NO), physiologically needed to maintain vascular homeostasis, tissue perfusion, and prooxidant/antioxidant activity^2,5^. Loss of ATP in aged RBCs is likely to have a negative impact on NO production in the endothelium thus disrupting vaso-regulatory homeostasis^6–8^.

In two separate experiments using sickle cell and old stored RBCs (which share some common features with sickle RBCs), we recently have reported that an internal Hb oxidation reaction intermediate, ferrylHb (HbFe^4+^), plays a key role in initiating RBC membrane changes^9–11^. Specifically, we found that ferrylHb directly targets band 3 and its associated proteins. The complex formation between the Hb and band 3 network of proteins that undergo extensive phosphorylation and ubiquitination lead in the case of sickle cell disease (SCD) blood (human and mice) to an increase in MP formation^9,11^.

Current research countermeasures that are designed to control the biochemical consequences of age-related changes in RBCs include targeting RBCs’ internal ROS generating sources and Hb oxidative side reactions^3^. Since changes are also manifested in membrane alterations, we looked for an additive that can target both internal oxidation reactions as well as membrane changes associated with the ageing process.

The anti-inflammatory and antiproliferative agent curcumin has been shown to be an effective antioxidant in the literature^12^. Specifically, the interaction of curcumin with the redox active iron in the membrane has been found to improve RBC deformability and minimize its degradation. However, its usefulness as an RBC protectant has been limited due to its poor solubility in both plasma and biocompatible solvents^12^.

The properties of curcuminoids (also referred to as “curcumin” herein) include both pro- and antioxidant activities, free radical scavenging, and impacting transcription and signaling mechanisms^13,14^. A major problem with using curcumin therapeutically is its low bioavailability after oral administration and attendant rapid metabolism^15,16^. A new curcumin formulation (VAS-101), manufactured by Vascarta Inc., is used in this study as an additive to stored blood/RBCs to assess its ability to enhance stability and storage time and to minimize the pro-inflammatory properties of aged RBCs^17^. This formulation is stable at least 2 years at ambient and accelerated conditions at a concentration of curcuminoids (0.1 M) that facilitates the addition of therapeutic amounts of curcumin within a small volume dose.

## Results

### Curcuminoid gel protects RBCs from oxidative stress during short-term storage.

**Figure 1:**
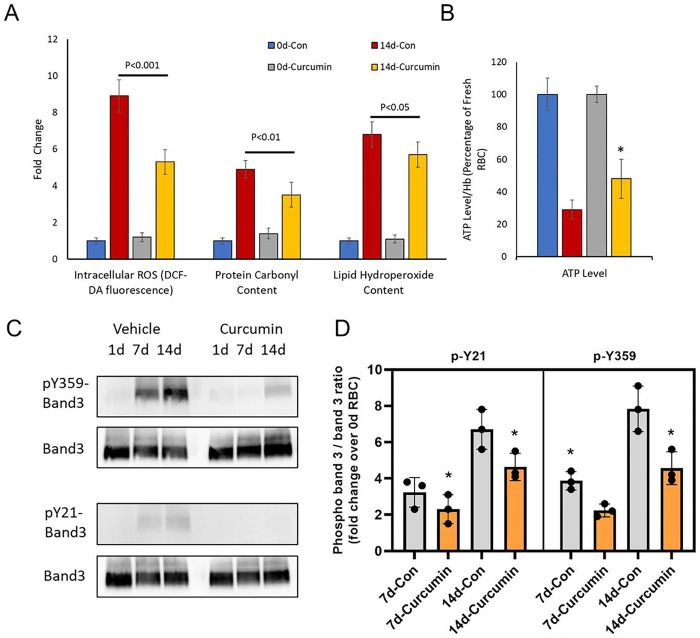
Curcumin protects RBCs by preventing oxidative stress and band 3 phosphorylation. (A) Histograms showing the effects of curcumin treatment on intracellular ROS formation, levels of protein carbonylation and lipid hydroperoxide formation in fresh RBCs (day 0) and in RBCs stored in DPBS (14 days). (B) ATP levels within day 0- and 14-day-old RBCs were also measured following treatment with curcumin. * P< 0.01 compared to 14-day control RBC (untreated). Total band 3 level and phosphorylation of band 3 in fresh RBCs (day 0) and 7- and 14-day-old (stored in D-PBS) RBCs were assessed by specific antibodies against band 3 or against specific phosphotyrosine residues (Y359 and Y21) as described in the methods (C). Histogram showing phospho band 3 / band 3 ratio, fold change over fresh (day 0) RBCs (D).

We investigated the antioxidant effect of a single-dose curcuminoids on RBCs stored in D-PBS for 14 days. Three (3) major parameters of oxidative change were studied, i.e., intracellular ROS, protein oxidation (carbonylation) and lipid hydroperoxide formation (Figure 1A). In untreated control groups, all 3 parameters were increased several folds after 14 days over the 0-day control. A single dose (500μM curcuminoids) at 0-day significantly reduced (~30–40%) all the oxidative damage markers. However, the most noticeable change was observed in intracellular ROS (~50% reduction) over 14 days compared to untreated control (Figure 1A). ATP measurements (bioenergetic indicator) in RBCs after 14-day incubation with curcuminoids led to almost a 40% recovery in PBS solutions (Figure 1B).

### Curcuminoids prevent band 3 phosphorylation.

We have previously documented that ferrylHb, once formed, can target several protein structures within RBC membranes, including band 3 proteins, causing oxidative and post-translational modifications, clustering of band 3, and MP formation^9,11^ (see supplementary Figure 1 for ferryl Hb measurements). We studied the effects of curcuminoids on ROS (Figure 1A), ATP (Figure 1B) and the phosphorylation of RBC membrane proteins at two common tyrosine phosphorylation sites (i.e., Y359 and Y21) using specific anti-phospho-tyrosine antibodies. These sites act as redox sensors of RBCs. Stored RBC samples in D-PBS were also analyzed for band 3 phosphorylation. Figure 1C and D show a gradual increase in band 3 phosphorylation at both tyrosine phosphorylation sites although no significant change in total band 3 levels were observed. A single-dose (500 μM) curcumin treatment at day 0 significantly reduced band 3 phosphorylation at day 7 and 14 (Figures 1C and D).

### Effects of curcuminoids on RBCs under long term (49 days) storage conditions.

**Figure 2:**
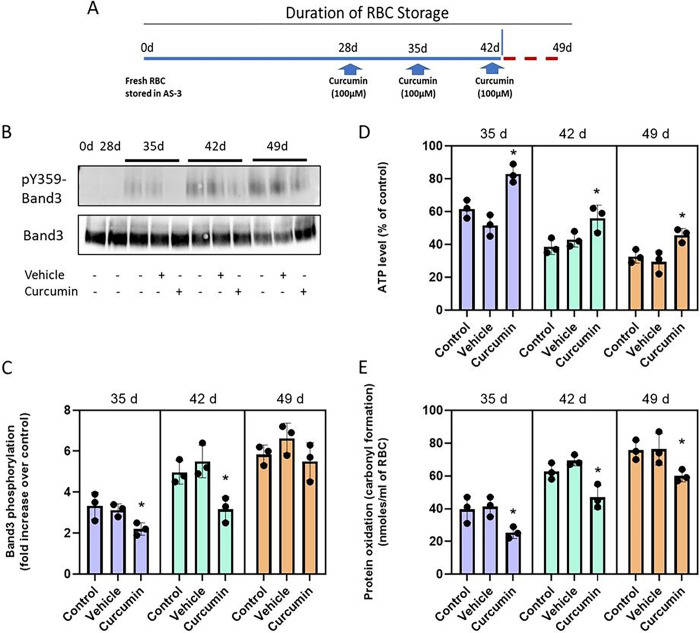
Effects of Curcumin on ATP and protein carbonylation over 49-day period. Scheme showing curcumin treatment of stored RBCs in AS-3 solution for up to 49 days (A). RBCs were stored in AS-3 solution for up to 49 days with or without curcumin or vehicle. The effect of curcumin treatment on RBCs was assessed by measurement of band 3 (Y359) phosphorylation (B, C); intracellular ATP levels (D); and levels of protein oxidation (E). Histograms show the comparison between vehicle and curcumin treatment. *P<0.01 vs corresponding vehicle treated group, N=3.

With the improvement in the redox milieu of RBCs in the presence of curcuminoids, we reasoned that the survival of RBCs for an additional week (49 days) may be feasible. We incubated RBCs in AS-3 solutions using the same previously used storage conditions for 49-day storage. We consecutively added 50μm curcuminoids at 35, 42 and 49 days as shown in the scheme (Figure 2A). Aliquots from each sample were taken at the end of each interval for analysis. Table 1 summarizes key measures of RBC viability with time. Oxygen affinity (P_50_), oxidation status (metHb) and hemolysis were measured for control samples, curcuminoids (in VAS-101), and in the vehicle (VAS-101 without curcuminoids). Biochemical parameters reported in Table 1 after incubation of curcuminoids with RBCs in all age groups to remain unchanged in keeping with previously reported values by our group^10^. This may suggest that curcuminoids were unable to penetrate the RBC membrane to directly interact with intracellular Hb. Although very little information is available in the literature on the direct interaction of curcuminoids with Hb, alterations in the conformation of Hb due to its reaction with curcuminoids was reported using UV absorption and CD spectroscopic methods. The α-helicity of Hb was found to decrease upon binding with curcuminoids. There was a small loss of α-helical secondary structure of Hb but no major functional changes were reported upon interaction with curcuminoids^18^.

Oxidation of the heme iron in intraerythrocytic Hb remained largely unchanged (below 1%) compared to controls at all age groups (35–49 days) in AS-3 solutions (Table 1). However, there was a slight increase in the levels of metHb in the vehicle plus curcuminoids (VAS-101) over the vehicle alone at 35, 42 and 49 days, respectively, reaching as high as 3% at day-49. The extent of hemolysis measured here as the percentage of free Hb in relation to the hematocrit (HCT) was 2.95% and 3% at 42 and 49-day incubation, respectively (Table 1). Generally, the United States Food and Drug Administration expects that hemolysis in stored RBC units should not exceed 1% at 42-days ^19^. It is noteworthy that the lower levels of hemolysis reported in the literature during similar storage conditions is likely due to the use of additive solutions such as SAGM (Saline, Adenine, Glucose, Mannitol) and DEHP (di-2-ethyl hexyl phthalate).^20^

### Effects of curcuminoids on RBCs band 3 phosphorylation under long term (49 days) storage conditions.

We then studied the effects of multiple doses of curcuminoids on the phosphorylation of RBC membrane protein band 3 when RBCs were stored for up to 49 days in AS-3 solution. Figures 2B, C show a consistent increase in band 3 phosphorylation (Y21) after 35 days. Vehicle treated samples did not show any changes when compared to untreated controls at 42 and 49 days, respectively. However, a significant decrease (20–30%) in band 3 phosphorylation was observed in 35-day and 42-day samples that were previously treated with curcumin. However, at 49 days, curcumin treatment did not show significant protection compared to the 35-day and 42-day timepoints.

### Effects of curcuminoids on ATP and protein carbonylation.

We then measured ATP content and protein oxidation in RBCs stored in AS-3 solution for up to 49 days in the presence of curcumin. As was seen in D-PBS solutions earlier, a rapid decline (from 40% at 35 days to 80% at49 days) in ATP was observed with a concomitant rise (up to six-fold) in protein oxidation as indicated by carbonyl content in RBCs during this same comparative timeframe. Weekly single-dose (100μM) curcumin additions, starting from day-28, provided considerable recovery (30–50%) in ATP and a significant drop (up to 50%) in carbonylation for all the time points studied (Figures 2D and E).

### Effects of curcuminoids on hemoglobin posttranslational modifications (PTMs) and RBC proteomics.

We monitored changes in oxidation of Hb by monitoring changes in oxidation levels of key amino acid residues of β-Hb subunit (W15, W37, M55, H78, C 93, C 112 and H117), known as the “hotspot” amino acids serving as reporters for oxidative changes in Hb^21^. We computed the ratio of oxidatively modified peptides to total peptides and reported the ratios in Table 2. A comparison of ratios for the different sample groups - 0 days (control), 35 days (control), and curcumin treated (35 days) shows that only a small percentage of the overall amino acid content underwent oxidation, typically 2% or less. M55 is particularly sensitive to oxidation and shows a modest 25% increase at 35 days with the same increase recovered following treatment with curcumin. Trioxidation C 93 has been extensively used by our group to monitor active site oxidation in Hb ^21,22^.There was no appreciable difference in oxidation of C93 as a result of ageing or treatment with curcumin.

**Figure 3:**
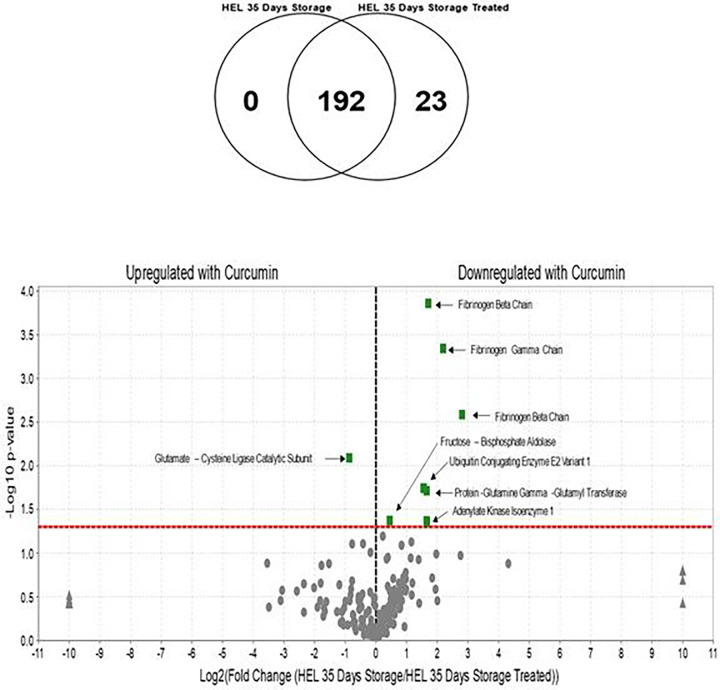
Protein distribution in RBC lysates and Volcano plots for the 35-day storage. (A): Venn diagram showing the distribution of proteins in the RBC lysates from curcumin treated and untreated control samples. The diagram shows that all the proteins in the 35-day group (192) are found in common with the 35-day treated group. There are 23 proteins that are exclusively found in the curcumin treated group. (B) Volcano plots (35 days control and 35 days treated with curcumin) show the ratio of the log 2-fold differences between biological categories where weighted spectral counts of Human Erythrocyte Lysates (HELs) stored for 35 days is the control relative to HELs stored for 35 days and treated with curcumin. Proteins that are upregulated or downregulated as a function of treatment with curcumin are shown as differences determined by plotting *p* values (−log_10_) for each protein against log-2-fold change (log_2_) difference. The *X*-axis shows the log of the fold change between the two timepoints. The *Y*-axis is the negative log of the *p*-value. Points above the red line represent significant changes. Points to the left of the *Y*-axis are enriched after 35 days, and points to the right are decreased at 35 days.

The Venn diagram in Figure 3A shows the difference in the number of proteins grouped according to the specific RBCs treatment. A total of 192 proteins were found across the samples, with 23 proteins exhibiting a statistically significant abundance change (p-value <0.05).

Quantitative comparisons of proteins between the 3 sample groups as illustrated in Figures 3B shows a remarkable increase of Fibrinogen subunits over a 35-day timeframe following curcumin treatment. Also noteworthy is the decrease in Fructose bisphosphate aldolase, the fourth enzyme in the glycolytic pathway, which catalyzes the conversion of fructose 1,6 bisphosphate into dihydroxyacetone phosphate and glyceraldehyde 3-phophate. Aldolase binds to the amino terminus of band 3. N-terminal 21 amino acid residues of band-3 are sufficient for binding and inhibiting the activity of the aldolase^23,24^. This may be linked to down regulation of ATP production in RBCs in the day 35 samples and the partial increase of ATP production following treatment with curcumin.

**Figure 4:**
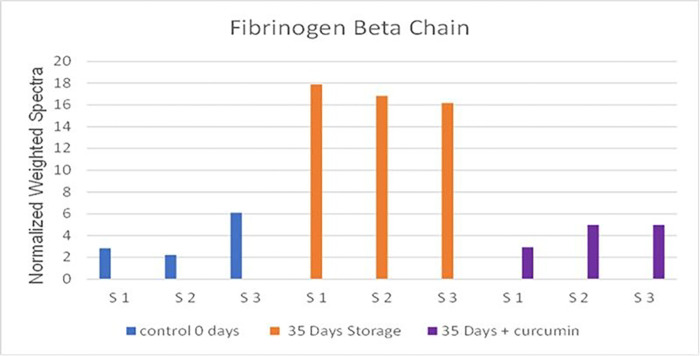
Fibrinogen Levels derived from mass spectrometric analytical runs. Fibrinogen levels are shown as a histogram of weighted spectral counts for fibrinogen β chain in individual mass spectrometry analytical runs where S1 – S3 represents the sample number. The data shows a clear increase in fibrinogen levels in the 35-day curcumin sample. Fibrinogen levels restored to day 0 levels following curcumin treatment with day 35 samples.

Ubiquitin conjugating enzyme E2 variant 1, protein glutamine gamma glutamyl transferase, and adenylate kinase isoenzyme 1, also show a decrease in concentration following treatment with curcumin. Ubiquitin conjugating enzyme E2 variant 1, although it has no Ubiquitin ligase activity, is thought to be involved in the formation of polyubiquitin chains that are not targeted by the proteasome machinery ^25^. Protein glutamine gamma glutamyl transferase is a transaminase that may be involved in protein cross linking, posttranslational modifications, or apoptosis. Adenylate kinase isoenzyme 1 is a ubiquitous enzyme that catalyzes the reversible transfer of the terminal phosphate group between ATP and AMP^26^. It catalyzes production of nucleotide triphosphates from the corresponding diphosphate substrates with either ATP or guanosine triphosphate (GTP). The one upregulated protein shown in the Volcano plots, glutamate cysteine ligase catalytic subunit, is the first rate-limiting enzyme of glutathione synthesis. Figure 4 shows fibrinogen Levels derived from mass spectrometric analytical runs, and it shows a particular increase in fibrinogen levels at day-35.

### Circulation lifetime of curcumin treated and untreated stored RBC’s.

**Figure 5:**
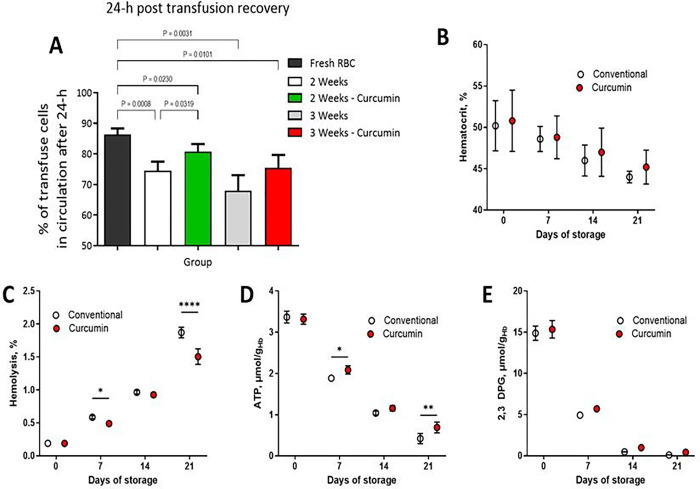
Post-transfusion recovery rates of RBCs and other bioenergetics and hematological induces in guinea pigs. Figure 5A illustrates the 24-hour post-transfusion recovery rates of RBCs stored conventionally and with curcumin supplementation over a storage period of 2 to 3 weeks. Figure 5B depicts the hematocrit levels over a storage period of 3 weeks. Figure 5C showcases the rates of hemolysis of stored RBCs over a period of 3 weeks. Figure 5D illustrates adenosine triphosphate (ATP) levels during storage. Figure 5E showcases 2,3-diphosphoglycerate (2,3-DPG) concentrations in stored RBCs. RBCs stored with curcumin exhibited statistically significant improvements in post-transfusion recovery compared to conventionally stored RBCs, particularly evident after both 2 and 3 weeks of cold storage. Notably, while conventionally stored RBCs at 3-weeks experienced a decline in recovery rates below 75%, RBCs stored with curcumin-maintained recovery rates of 81% and 75%, respectively, at 2 and 3 weeks of storage, highlighting the potential benefits of curcumin supplementation in storage.

RBCs stored with curcumin demonstrated superior 24-hour post-transfusion recovery rates compared to conventionally stored RBCs, particularly evident after 2 and 3 weeks of storage. Notably, after 3 weeks, while conventional storage led to a 75% decline in 24-hour post-transfusion recovery, RBCs stored with curcumin-maintained recovery rates above this critical threshold (Figure 5A), underscoring the benefits of curcumin supplementation in the storage medium. Moreover, throughout the refrigerated storage period, curcumin-treated RBCs exhibited higher hematocrit levels (Figure 5B) and lower rates of hemolysis compared to conventionally stored counterparts, mainly notable after 3 weeks (Figure 5C). ATP and 2,3-DPG levels naturally declined over time; however, curcumin demonstrated a remarkable capacity to mitigate this decline. Although the absolute effects on ATP preservation may not be physiologically significant, RBCs stored with curcumin consistently exhibited higher ATP levels compared to conventionally stored RBCs, with a notable 35% difference after 3 weeks storage (Figure 5D), consistent with our *in vitro* studies (Figure 1B). Similarly, while the direct physiological relevance of curcumin’s impact on 2,3-DPG levels is unclear, its ability to enhance 2,3-DPG concentrations during storage showed increases of 10%, 50%, and 75% observed at 1, 2, and 3 weeks, respectively, compared to conventionally stored RBCs (Figure 5E). Despite these profound biochemical effects, no significant correlation was observed between 24-hour post-transfusion recovery and ATP, 2,3-DPG, hemolysis, or hematocrit levels, highlighting the multifactorial nature of post-transfusion outcomes. The significant improvements in storage parameters conferred by the curcumin additive emphasize its role in enhancing the quality, stability and efficacy of stored RBCs.

## Discussion

Cold storage of blood results in a multitude of biochemical and physiological changes collectively known as the “storage lesion”. Impaired nitric oxide (NO) metabolism, increased lactic acid levels, decreased pH, ATP, and 2,3-diphosphoglycerate (2,3-DPG), the release of inflammatory mediators, and increased red cell membrane inflexibility are among the most cited adverse changes ^2,27^. Membrane vesiculation, MP formation, Hb’s oxidative modifications and progressive loss of cellular homeostasis and antioxidant defenses are some of the other metabolic and structural changes that occur within RBCs during prolonged storage^28^. Post-translational modifications (PTMs), such as phosphorylation, protein oxidation, and aggregation are functionally involved in the regulation of RBC homeostasis and lifespan^3^. Stored blood (RBCs) can cause many adverse safety events in transfused patients (e.g., Transfusion-Associated Circulatory Overload (TACO) and Transfusion-Related Acute Lung Injury (TRALI)). Both are syndromes of acute respiratory distress that occur within 6 hours of transfusion. TACO and TRALI are the leading causes of transfusion-related fatalities ^29^.

Although the storage lesion has been well-documented for decades^30^, our understanding of the mechanisms involved in these changes and the clinical consequences remains incomplete. The progression of the storage lesion was shown recently to be driven in part by internal redox oxidative reactions mainly of Hb, hence the process is also termed “oxidative lesion” ^3,10^. Hb’s iron oxidation affects not only the ability of RBCs to carry oxygen but also these reactions could be damaging to the RBCs and surrounding tissues. Hemolysis is another consequence of Hb oxidation. It has been suggested that antioxidants should be added to reduce hemolysis during the cold storage of blood^31^ . Factors affecting the rate of Hb oxidation during RBC storage include compromised antioxidant activity, high concentrations of glucose in the storage media, and the presence of molecular oxygen^32^. Most recently, we showed that oxidation of the heme iron plays a pivotal role (with time) in creating an oxidative milieu within the cytosol of RBCs that can impact membrane proteins, including band 3.

The most common intervention strategies designed to control the biochemical consequences of RBC age-related changes include the targeting of (i) the source(s) of ROS by subjecting RBCs to periods of hypoxia in hypoxic chambers^33^, (ii) Hb oxidation with reductants such as ascorbic acid or caffeic acid^10,34–36^ and (iii) RBC membranes, specifically band 3 complex proteins ^37^. Individual or any combination of these methods may minimize biochemical changes and restore oxygen homeostasis in tissues.

A topical formulation, VAS-101, was developed by Vascarta Inc. (Summit, NJ, USA) to transdermally deliver bio-active concentrations of curcuminoids, an NO promoter, anti-inflammatory and pain-relieving agent. These curcuminoids were recently shown to mitigate endotoxemia by modulating endothelial NO due to modulation of the endothelium and/or an indirect anti-inflammatory action^17^. VAS-101 has also effectuated a significant reduction in inflammation, mast cell activation, and hemolysis, and metabolic stabilization of sickle RBCs in a humanized SCD mouse model (GoelY. et al., Manuscript under revision, Blood Advances). We investigated the interaction of this novel curcumin gel formulation with stored RBCs that are known to undergo cytosolic and membrane changes with time.

Oxygen binding parameters were obtained in the current study at the 35-to-42-day timeframe, consistent with our previous data and with that of others for stored blood^10,38^. Adding curcuminoids produced no changes in either the P_50_ (~18 mmHg) or in the cooperativity of Hb (~2.0). This rules out direct binding of curcumin with intraerythrocytic Hb.

Iron oxidation of intraerythrocytic Hb at 4°C shows a typical slow process of autoxidation and metHb formation during the 42-day incubation (Table 1). However, as we have shown recently, this process is exacerbated at 37°C when a larger quantity of metHb is accumulated^10^. Ferryl Hb, once formed under oxidative stress conditions, tends to accumulate at higher levels in young RBCs rather than older ones which may be due to a more robust pseudoperoxidase activity in younger RBCs^39^. Additionally, because of its powerful oxidizing ability, ferryl Hb targets cytosolic and membrane proteins such as membrane band 3 proteins^9,40^. As we have shown, this leads to band 3 clustering and MP formation. A critical step in this process is the phosphorylation of key tyrosine residues. Our data shows that curcumin suppressed phosphorylation of tyrosine residues (Y359 and Y21) (~30%) without altering band 3 proteins.

Other markers of oxidative stress, such as ROS, protein carbonyl content and lipid hydroperoxide, were similarly suppressed when young (day 0) and day 14 RBCs were treated with curcumin. In our experiments with curcumin there was almost a 33% recovery in ATP in RBCs following 35-days which then began to slowly to decline over the next 2 weeks. It has been suggested that RBC ATP export tends to increase in response to hypoxia or deformation in the microvasculature leading to blood vessel dilation. By augmenting RBC ATP in some disease states or for use in blood banking, it may result in an improvement in RBC function^41^.

Proteomic analysis of RBC lysates from two groups (d0 and day 35) confirmed the presence of protein alterations, including the downregulation of both bands 3 and 4.1, consistent with our recent proteomic analysis of young and older RBCs (42-days)^10^. The most impressive findings in our current proteomic analysis of RBCs treated with curcumin is the remarkable drop in Fibrinogen subunits. Fibrinogen as a plasma protein (a dimeric molecule composed of pairs of α, β, and γ chains that are folded into a three-domain nodular structure) is involved in regulating blood viscosity in circulation as well as crucially serving as a controlling mechanism in blood clotting ^42,43^.

**Figure 6:**
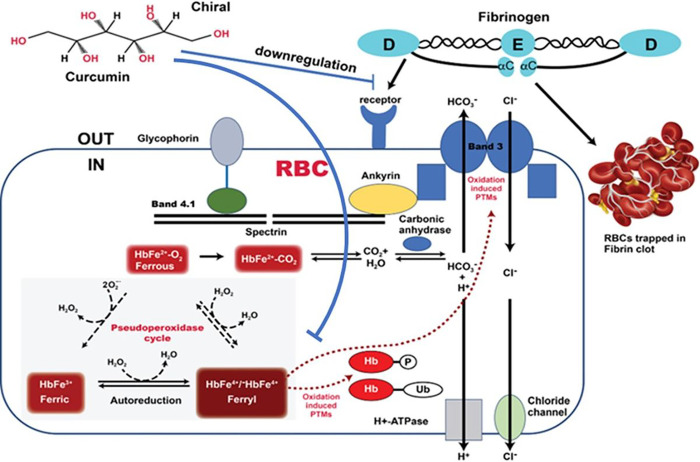
Curcumin downregulates fibrinogen and prevents RBC aggregation. Proposed model for the interaction of curcumin with fibrinogen to prevent aggregation of aged RBCs. Membrane bound band 3 and its network of structural proteins provide efficient anion exchange of bicarbonate (out) in exchange for chloride (in). Hemoglobin plays a critical role in the removal of (CO2) (~80%) by converting it to bicarbonate catalyzed by the enzyme carbonic anhydrase. Hemoglobin-dependent conformation transition (deoxy/oxy) in which the deoxy form of Hb interacts directly with band 3 proteins has been shown to regulate glycolysis in RBCs ^54^. A redox transition during oxidation of hemoglobin into a higher oxidation form (ferrylHb), through its pseudoperoxidase cycle, interacts with band 3 resulting in oxidative modifications of band proteins in both old and diseased RBCs. Fibrinogen at higher concentrations promotes RBC aggregation through binding to a receptor on the RBC membrane. Curcumin inhibits ferryl Hb formation and downregulates fibrinogen to prevent aggregation. Adapted with modification from Jay, Cell 1996 ^54^ and Jana et al.2018 ^9^.

It is well established that fibrinogen-induced RBC aggregation and the adsorption of fibrinogen macromolecules onto RBC membrane leads to cell bridging during intercellular interactions resulting in the formation of RBC aggregates called “rouleaux” ^43^. Higher levels of fibrinogen are known to increase RBC deformability due to dephosphorylation of band 3 tyrosines. For the binding between fibrinogen and RBCs to occur, a lower fibrinogen concentration is needed in young RBCs compared to older RBCs^42^. This may provide a mechanistic insight into the mode of action of curcumin in blood (Figure 6) since high levels of fibrinogen in blood promote RBC aggregation by binding to specific RBC receptors on membranes of these cells. Curcumin in our study downregulated fibrinogen and prevented RBC aggregation.

Preclinical models offer a unique opportunity to examine possible harmful effects of using RBC units nearing their storage expiry for transfusion that may not be readily available from human trials. Although preclinical studies do not provide predictions of the risk in actual clinical transfusions, they provide an understanding of the causes behind transfusion-related problems, and how RBC storage time, the number of RBC units used, and the patient’s overall clinical condition, might be interconnected^1,44^.

The rationale for the use of guinea pigs is based on the similarity between guinea pigs and humans regarding their overall antioxidant status. Like humans, guinea pigs cannot produce their ascorbic acid (AA) due to the loss of a liver enzyme called l-gluconolactone oxidase (LGO). Both guinea pigs and humans appear to compensate for the lack of AA production by increasing the content and efficiency of antioxidant enzymes in their tissues. For instance, the activity of superoxide dismutase enzymes in the kidney and liver is about twice as high in humans and guinea pigs compared to rats^45^. Recent research has shown that like humans, guinea pigs, but not rats, have a similar system for recycling prooxidant dehydroascorbate (DHA) back into functional AA within their RBCs^46^. This indicates a similar antioxidant status between guinea pigs and humans, with a greater emphasis on tissue rather than plasma antioxidant capacity.

The 24-hour post-transfusion recovery, is a metric utilized to gauge the quality of stored RBCs, is based on the principle that RBCs must circulate sufficiently, post-transfusion, ^47^. This parameter uses a minimum of 75% of transfused RBCs circulating for at least 24 hours. Assessing 24-hour post-transfusion recovery which involves infusing a small volume of radiolabeled cells and measuring surviving labeled RBCs after 24 hours. are currently under investigation ^48^.

Curcumin-stored RBCs exhibited 24-hour post-transfusion recovery rates of 81% and 75% after 2 and 3 weeks of storage, respectively, contrary to conventionally stored RBCs that had 74% and 69% recovery rates after similar storage periods.

In a recent large metabolomic study, blood from 8,502 healthy blood donors stored for 42 days was studied to determine the propensity of the RBCs to hemolyze following oxidant stress using the oxidative hemolysis agent, 2,2’-azobis-2-methyl-propanimidarochloride (AAPH, 150 mmoL). Alterations in RBC energy and redox homeostasis were more common in donors with high oxidative hemolysis. The authors suggested that the introduction of dietary antioxidant supplements prior to donation in recurring donors should be considered^49^. VAS-101, a novel curcumin-based gel formulation, has been shown here to specifically restore oxygen homeostasis and mitigates (or delay) the advent of the storage lesion in stored human blood. These findings strongly suggest the utility of VAS-101 as a potential additive (reagent) that could be used in the biopreservation and reversal of oxidative injury in human blood during storage.

## Experimental Procedures

### Blood collection and storage of RBCs:

Blood samples used in this study were obtained from healthy donors of age at least 18 years or above with written informed consent from the National Institute of Health (NIH) Blood Center, Bethesda, Maryland (FDA/CBER, IRB protocol 03084B) [amendment 03–120B (for red cells)]. All research performed in this study are in accordance with relevant guidelines/regulations approved by Research Involving Human Subjects Committee (RIHSC 2021-CBER-041). For RBC storage, approximately 100 ml of whole blood was first passed through a neonatal high efficiency leukocyte reduction filter and then centrifuged at 2500 X g for 10 mins to separate RBCs from platelets rich plasma. After removal of plasma and top-buffy coat, packed RBCs were mixed with 22 ml of AS-3 storage solution and then stored at 4°C in a 100 ml capacity neo-natal red-cell storage bag (Neo Bag) for up to 49 days following a standard blood banking protocol. For some short-term experiments, RBCs were also stored in a dextrose (1gm/L) containing PBS (DPBS). Packed RBCs were mixed with equal volume of DPBS after removing the plasma and stored in 100 ml Neo bags for up to 14 days.

### Treatments of RBCs with curcumin-gel formulation:

In this study, we used VAS-101, a novel non-aqueous, biocompatible gel formulation (Vascarta Inc, Summit, NJ, USA), containing a very high concentration of curcuminoids (0.1 M). The curcuminoids (containing high levels of all three naturally occurring curcuminoids) were obtained from a proprietary turmeric root extract (Curcugen^®^, Dolcas-Biotech Inc, Landing, NJ, USA) dissolved in a PEG 400 based solvent to promote high solubility and stability. The gel formulation without curcuminoids served as the vehicle. Both VAS-101 and the vehicle were developed and provided by Vascarta Inc.

For the short-term study in DPBS, 500 μM curcumin was added to RBCs in storage bags on the first day (0d) of storage. The same volume of a vehicle was added to another bag as the non-treatment control. For the longer-term storage, curcumin (100 μM) or vehicle were added to respective storage bags on day 28, 35, 42 and 49, respectively.

### Spectrophotometric analysis:

1ml of the stored blood was taken at day 1, 7 and 14, respectively, and incubated for 1 hour at 37 °C, and then equilibrated at room temperature. The solution was then washed with 2 ml PBS, gently stirred, and then centrifuged for 5 minutes. The supernatant was removed. This process was repeated twice. The RBCs were lysed by adding 3ml of water, gently stirred and left to stand for 10 minutes at room temperature. NaCl (24mg) was added to the lysate and the mixture was centrifuged at 4000×g for 10 minutes. The supernatant was filtered with 0.2 μM to remove RBC membranes. The solution was concentrated and the Hb concentration was measured^50^.

### Autoxidation and hydrogen peroxide-mediated oxidation of RBCs

Spontaneous oxidation of RBCs (60μM/heme) taken from the stored blood in AS3 solution in the presence/absence of curcumin (100 μM) was measured spectrophotometrically for 35 days. Spectral measurements were captured at room temperature for 24 hours. A stock solution of 60 μM of Hb (per heme) was prepared for oxidation experiments. The Hb solution was treated with 20 mM H_2_O_2_ for 5 minutes followed by an immediate addition of 2mM Na_2_S to capture transient ferryl Hb. Spectra were captured at each stage of the Hb transformation (oxyHb, metHb, ferrylHb and sulfHb). Extinction coefficients for each species were used as previously reported by our group^50^.

### Gel electrophoresis and immunoblotting:

Cell lysate proteins were resolved by SDS-PAGE using precast 4–20% NuPAGE bis-tris gels (Thermo Fisher Scientific, Waltham, MA, USA) and then transferred to nitrocellulose membranes (BioRad, Hercules, CA, USA) using standard immunoblotting techniques. Nitrocellulose membranes were processed with different specific primary antibodies [e.g., anti-β actin (ab8227), anti-band3 (ab108414), anti-phospho Y359 band 3 (ab77236) and anti-phospho Y21 band 3 (ab125070) (Abcam, Cambridge, MA, USA)]. Appropriate HRP-conjugated goat anti-mouse IgG (ab97040) and anti-rabbit IgG (ab205718) secondary antibodies were also obtained from Abcam (Cambridge, MA, USA).

### Measurement of ROS, ATP and protein carbonylation in aged RBCs:

Measurement of ROS in RBCs were carried out fluorometrically using a cell-permeant fluorometric probe 2’,7’-dichlorofluorescin diacetate (DCFDA) that detects different reactive oxygen species (ROS) including hydroxyl, peroxyl radicals ^51^. Upon oxidation, fluorescent DCF was detected by fluorescence spectroscopy with excitation/emission at 495 nm / 530 nm (26). Briefly, RBCs (995 μL 10% v/v suspension in PBS) were incubated with 5 μL of DCFDA (10 mol/L) at 37 °C for 30 min. Following the incubation, the suspension was further diluted 20 times in PBS and the fluorescence was measured using a Synergy-HTX 96-well plate fluorimeter (Biotek Instruments, Winooski, VT, USA). ROS formation was expressed as relative fluorescence units (RUF)/mg Hb.

Intracellular ATP levels in fresh and stored RBCs were measured using a colorimetric ATP-assay kit from Sigma-Aldrich (Sigma-Aldrich, St. Louis, MO, USA) following a method previously published^51,52^. First, fresh or stored RBCs were washed and then resuspended in PBS containing 1% glucose, 170mg/L adenine and 5g/L mannitol for the ATP measurement. ATP concentration was determined by phosphorylating glycerol, resulting in a colorimetric (570 nm) product proportional to the amount of ATP present using a commercial kit from Sigma-Aldrich (Sigma-Aldrich, St. Louis, MO, USA).

Protein carbonyl content in RBC lysates was assessed by a dinitrophenyl hydrazine (DNPH) based assay kit (ab126287) as a measure of protein oxidation (Abcam, Cambridge, MA, USA). In these experiments, carbonyl groups in protein side chains are derivatized to DNP-hydrazone following reaction with DNPH. The absorbance of DNP hydrazones formed in this reaction were measured at 375 nm using a BioTek Synergy HTX microplate reader (Agilent, Santa Clara, CA, USA).

### Statistical analysis:

Plotting of raw data and all statistical calculations were done using GraphPad Prism 8 software. All values are expressed as mean ± SD and error bars in the bar diagrams are indicative of SD. A p-value of <0.05 was considered statistically significant. The difference between two means were compared using paired Student’s t-test.

### Proteomic analysis of RBCs stored for 35 days:

#### Sample preparation:

Protein extraction from RBC lysates was done using lysis buffer (8M urea, 50 mM Tris HCl pH 8.0, 150 mM NaCL, 1x Roche Complete protease inhibitor). Sonication was done using a QSonica sonic probe with the following settings: Amplitude 50%, Pulse 10 × 1s, 1 on and 1 off. The lysate was then incubated at room temperature for 1 hr with mixing at 1,000 rpm in an Eppendorf Thermomixer. The lysate was clarified by centrifugation at 10K g for 10 min at 25°C.

#### Proteolysis of extracted protein:

20 μg of each sample was reduced with 14 mM dithiothreitol at 25°C for 30 minutes followed by alkylation with 14 mM iodoacetamide at 25°C in the dark. Proteolysis was done using 2.5 μg trypsin (Promega sequencing grade) at 37°C overnight. The proteolyzed samples were cooled to room temperature. The volume of the sample was brought to 0.5 ml with ammonium bicarbonate. The proteolyzed samples were centrifuged at 10,000 x g and desalted using a Waters HPB solid phase extraction plate. Samples were lyophilized and reconstituted with 0.1 % TFA prior to MS analyses.

#### Mass spectrometry:

Mass spectrometry experiments were carried out at Bioworks Laboratories (Ann Arbor, MI, USA). The equivalent of 1μg of each digest was analyzed by nano LC-MS/MS with a Waters NanoAcquity HPLC system interfaced to a ThermoFisher Fusion Lumos mass spectrometer. Peptides were loaded on a trapping column and eluted over a 75μm analytical column at 350 nL/min with a 2hr reverse phase gradient; both columns were packed with Luna C18 resin (Phenomenex). The mass spectrometer was operated in data-dependent mode, with the Orbitrap set at a resolution of 60,000 FWHM and 15,000 FWHM for MS and MS/MS, respectively. The instrument was run with a 3s cycle for MS and MS/MS. Advanced Precursor Determination (APD) was employed.

#### Proteomic data analysis:

Raw files from the mass spectrometric analysis were converted to .mgf file format prior to searching against the Swiss Prot Database for protein identification. The database search was done with the following parameters: two missed cleavages, peptide tolerance 10 ppm, MS/MS tol. +/− 0.1 Dalton, variable modification (C) carbamidomethylation, (M) oxidation, (M) deoxidation, (C) trioxidation, (H W) oxidation, peptide charge = 1+,2+,3+. The data files from Mascot were then submitted to Scaffold for peptide and protein validation using “Peptide Prophet” and “Protein Prophet”. Probabilities were set to 95% for peptide identification and 90% for protein identifications. Label free quantitation was done using Scaffold’s “weighted spectral counting method”. The Volcano plot was generated by Scaffold 5 (mass spectrometry software).

Post Translational Modifications (PTMs) were identified by searching the initial results obtained from the Mascot Search of the raw mass spectrometry data using an error tolerant search. The results of the error tolerant search were then searched using Scaffold v 5 for file conversion and peptide and protein validation. These results were then submitted to Scaffold PTM for validation of the PTM assignment, quantitation and statistical analysis.

### Circulation lifetime and post-transfusion recovery of curcumin treated RBCs:

#### Animal preparations:

Animal handling and care followed the National Institutes of Health Guide for the Care and Use of Laboratory Animals, and the University of California San Diego Institutional Animal Care and Use Committee approved the experimental protocol. All methods were carried out in accordance with the ARRIVE guidelines (Animal Research: Reporting of In Vivo Experiments). Guinea pigs weighing between 300–400 g were used in this study.

#### Blood collection and preparation:

Guinea pigs were anesthetized with isoflurane (Drägerwerk AG, Lübeck, Germany) in compressed room air (flow rate 1.0 LPM) slowly, by increasing the isoflurane 0.4% every 3 min until a surgical depth of anesthesia was achieved, typically 3%. Under anesthesia, a femoral artery catheter was implanted. Each donor bled freely into 1.4 mL of CP2D taken from an AS-3 blood preparation kit (Haemonetics Corporation, Braintree, MA, USA) until 50% of blood volume was lost. Donor blood was then pooled, and CP2D concentration was adjusted to 14%. Pooled blood was centrifuged at 1000 g for 7 minutes, and the supernatant removed. AS-3 (22%/vol) was then added, and the blood was mixed gently by inverting the bag for 1 minute. Pooled blood was then passed through a neonatal leukocyte reduction filter (Haemonetics Corporation, Braintree, MA, USA). RBC units intended for storage received 500 μM curcuminoids on the first day (0d) of storage. The same volume of a vehicle (no curcuminoids) was added to another bag to serve as the vehicle control. At the 2nd and 3rd weeks of storage, blood was radiolabeled with Technetium-99 (Tc99) as described by Zink et. al^53^. Briefly, RBC samples (1.0 mL) were added to a sterile reaction vial and gently mixed to dissolve the lyophilized UltraTag-RBC (UltraTag-RBC, Mallinckrodt, St. Louis, MO, USA), and allowed to react for 5 to 7 minutes. Then, UltraTag pH buffers were added to adjust pH, by gently mixing them into the reaction vial and by inverting the container. Samples were washed with sterile PBS twice and centrifuged to remove unreacted Tc99, and labeled RBCs were injected. 200 μL of Tc99 radiolabeled blood (approximately 2% of blood volume) was delivered I.V. to male anesthetized guinea pigs and 65 μL samples were drawn at 5 minutes, 30 minutes, and 24 hours post-injection. Samples were all run for detection of radioactivity on a Cobra II gamma counter (Packard Instrument Co., Meriden, CT, USA) at the same time so that the reported counts were independent of sample time and only representative of the still-circulating radio-labeled RBCs.

ATP and 2,3DPG: Aliquots of RBCs were mixed with cold trichloroacetic acid (DiaSys Deutschland, Flacht, Germany) and vortexed for 60 seconds. Aliquots sat on ice for 5 minutes and were then centrifuged at 3600g at 4°C for 10 minutes. The supernatant was removed and frozen at −80°C for later analysis. Supernatants were assayed enzymatically with commercially available kits. DPG was measured with the Roche 2,3-Diphosphoglycerate kit (Roche Diagnostics, Indianapolis IN, USA) according to manufacturer’s instruction. ATP was measured by DiaSys ATP Hexokinase FS kit (DiaSys Diagnostic Systems GmbH, Holzheim, Germany) according to manufacturer’s instruction.

## Figures and Tables

**Table 1:** Oxygenation, oxidation reactions and hemolysis in RBCs treated with curcumin

Sample	35 Days				42 Days				49 Days			

	*Hemolysis*	Met %	P50	n	*Hemolysis*	Met %	P50	n	*Hemolysis*	Met %	P50	n
Control (AS3)	1.42	0.78	18.16	2.13	1.62	0.87	18.28	2.32	1.75	0.06	18.16	2.3
Cur (AS3 + Curcumin + Vehicle)	1.72	1.35	18.33	2.11	2.21	2.95	18.66	2.37	3	3.65	18.42	2.35
Veh (AS3 + Vehicle)	1.3	0.01	18.88	2.27	1.77	2.11	18.90	2.31	3.65	1.75	18.88	2.28

**Table 2:** Summary amino acid posttranslational modifications in aged RBCs treated with curcumin.

Site	Modification	Best Ascore	Localization	ratio control	35 days	35 days c
H3	Oxidation	1000	1	0.01	0.02	0.01
W16	Trp->Kynurenin	1000	1	0.01	0.02	0.01
W16	Oxidation	83.88	1	0.12	0.13	0.13
W16	Quinone	1000	1	0.01	0.01	0.01
W16	Dioxidation	80	1	0.05	0.05	0.04
W38	Trp->Kynurenin	1000	1	0.01	0.02	0.02
W38	Oxidation	60.65	1	0.02	0.04	0.02
W38	Dioxidation	35.92	1	0.03	0.04	0.04
T39	Oxidation (T)	1000	1	0.13	0.04	0.1
M56	Oxidation	83.61	1	0.17	0.19	0.17
F72	Oxidation	75.36	1	0.2	0.02	0.02
H78	Oxidation	63.52	1	0.01	0.02	0.02
C94	Trioxidation	1000	1	0.01	0.01	0.01
C94	Sulfur Dioxide	1000	1	~0	0.01	0.01
C113	Tri oxidation	1000	1	0.01	0.01	0.01
C113	SulfurDioxide	1000	1	~0	0.01	0.01
H118	Oxidation	44.44	1	~0	0.01	0.01

## Data Availability

The datasets used and/or analysed during the current study available from the corresponding author on reasonable request.

## Key Points

- RBCs stored for extended periods undergo biochemical and physiological changes described as the storage lesion.
- Curcumin reversed oxidative changes and prolonged RBC survival beyond 42-day storage and extended RBC circulation life in a guinea pig model
